# 826 Analysis of National Burn Repository: Impact of Dermal Templates in Burn Management 2008 to 2021

**DOI:** 10.1093/jbcr/iraf019.357

**Published:** 2025-04-01

**Authors:** Roselle Crombie, Claire Witherel

**Affiliations:** Connecticut Burn Center, Bridgeport Hospital / Yale-New Haven Health; Integra LifeSciences

## Abstract

**Introduction:**

Thermal burn injuries comprise of over 400,000 emergency room visits in the

United States each year, with estimated costs of care being over $1 billion. Dermal templates continues to be the only skin substitute on the market indicated for the management of third degree burns. Since introduction over 25 years ago, over 75 unique commercially synthetic and non-autologous tissue substitute products are available today. Many of these technologies have been used for patients where sufficient autograft was not available due to their physiological condition. The goal of this study was to elucidate how Integra’s dermal templates have been used over the last 14 years and aim to understand where it has the most utility in the burn community.

**Methods:**

National Burn Registry data from 2008-2021 were analyzed (n = 388,775 patients). Surviving patients treated with dermal regeneration template were specifically identified in ‘Resource Utilization’ of the dataset (n = 637 patients with dermal regeneration template alone, and n=751 patients had dermal templates in conjunction with other therapeutics, including allograft, xenograft, biological wound covering or multiple combinations). Aggregatedmetrics included patient demographic information (age, sex, comorbidities, burn location/area) and outcome measurements (LOS, total body surface area (TBSA) (2 nd, 3 rd, and combined), complications, resources, number of procedures, number of excisional and non-excisional debridements). Additional analyses included normalizing patients’ LOS per TBSA.

**Results:**

Preliminary analysis showed patients with a majority third-degree burn 40-59% TBSA and & >60% TBSA and treated with dermal template were associated LOS/TBSA of approximately 1.53 ± 0.74 and 1.06 ± 0.60, respectively. In 2021 alone, dermal template was associated with fewer infections compared to a temporizing matrix for all surviving burn patients regardless of burn depth.

**Conclusions:**

This study demonstrates the utility of National Burn Repository to study burn care algorithm and associated clinical performance. Future analysis will propensity match patients to investigate clinical and health economic implications on these cohorts.

**Applicability of Research to Practice:**

The NBR remains and excellent source for us to study our patients nationwide over time. Gaining insights from it’s analysis can help guide us as to what practices lead to improved outcome.

**Funding for the Study:**

Funding of the B-Data access was done by Integra Life Sciences. No funding was used for the analysis, research or this ongoing project.

